# Assessing the effect of a regional integrated care model over ten years using quality indicators based on claims data – the basic statistical methodology of the INTEGRAL project

**DOI:** 10.1186/s12913-022-07573-7

**Published:** 2022-02-24

**Authors:** Dominikus Stelzer, Erika Graf, Ingrid Köster, Peter Ihle, Christian Günster, Patrik Dröge, Andreas Klöss, Claudia Mehl, Erik Farin-Glattacker, Max Geraedts, Ingrid Schubert, Achim Siegel, Werner Vach

**Affiliations:** 1 Institute of Medical Biometry and Statistics, Faculty of Medicine and Medical Center, University of Freiburg, Freiburg, Germany; 2 PMV research group at the Department of Child and Adolescent Psychiatry, Psychotherapy and Psychosomatics, University of Cologne, Köln, Germany; 3 Health Services and Quality Research, Research Institute of the Local Health Care Funds (WIdO), Berlin, Germany; 4 Institute for Health Services Research and Clinical Epidemiology, University of Marburg, Marburg, Germany; 5 Institute of Occupational and Social Medicine and Health Services Research, University Hospital Tübingen, Tübingen, Germany; 6 Basel Academy for Quality and Research in Medicine, Basel, Switzerland

**Keywords:** Integrated care model, Evaluation, Quality indicator, Statistical analysis

## Abstract

**Background:**

The regional integrated health care model “Healthy Kinzigtal” started in 2006 with the goal of optimizing health care and economic efficiency. The INTEGRAL project aimed at evaluating the effect of this model on the quality of care over the first 10 years.

**Methods:**

This methodological protocol supplements the study protocol and the main publication of the project. Comparing quality indicators based on claims data between the intervention region and 13 structurally similar control regions constitutes the basic scientific approach. Methodological key issues in performing such a comparison are identified and solutions are presented.

**Results:**

A key step in the analysis is the assessment of a potential trend in prevalence for a single quality indicator over time in the intervention region compared to the corresponding trends in the control regions. This step has to take into account that there may be a common - not necessarily linear - trend in the indicator over time and that trends can also appear by chance. Conceptual and statistical approaches were developed to handle this key step and to assess in addition the overall evidence for an intervention effect across all indicators. The methodology can be extended in several directions of interest.

**Conclusions:**

We believe that our approach can handle the major statistical challenges: population differences are addressed by standardization; we offer transparency with respect to the derivation of the key figures; global time trends and structural changes do not invalidate the analyses; the regional variation in time trends is taken into account. Overall, the project demanded substantial efforts to ensure adequateness, validity and transparency.

**Supplementary Information:**

The online version contains supplementary material available at (10.1186/s12913-022-07573-7).

## Background

The integrated health care model “Healthy Kinzigtal” was established in 2006. The model addresses the full spectrum of morbidities and health issues for a population defined by a residential area (with the only exception of dental care). It is based on a contract among a regional physicians’ network, a management and holding company specialised in integrated care, and the Allgemeine Ortskrankenkasse Baden-Württemberg (AOK-BW), the largest statutory health insurance fund in the federal state of Baden-Württemberg (BW). The contract is a so-called shared savings contract, that is, the healthcare cost savings achieved are distributed between the contractual partners. (For further details we refer to the study protocol [1] and the main project publication [2].) From an economic perspective the contract was rather successful, as cost savings were achieved. However, a shared savings contract may lead to lower levels of care, that is, an underutilisation of health services. Consequently, an evaluation of the health care quality of the model is highly relevant. The aim of the INTEGRAL project was to conduct such an evaluation.

Comparing quality indicators based on claims data between the intervention region and 13 structurally similar control regions constitutes the basic scientific approach. More precisely, the idea was to determine for a single indicator the prevalence trend in each region and to compare the observed trend in the intervention region with the trends in the control regions. The control regions allowed not only a determination of the expected trend in the absence of the intervention. They also allowed the determination of the natural variation of these trends, i.e. we did not a priori assume that the trends are equal across the control regions. Regional differences in health care are well established in Germany [3], and it cannot be ruled out that there are also regional differences in the prevalence trends of quality indicators, reflecting ongoing changes in the local situation. Hence the fact that a trend in an intervention region differs from a mean trend in the control regions does not necessarily indicate an effect of the intervention. Such differences may also happen by chance, and therefore an evaluation has to take into account the variation in trends observed in the control regions. We hence aimed to conclude a specific role of the intervention only in the case that the observed trend in the intervention region could be regarded as an extreme one relative to the variation observed in the control regions.

The study protocol [1] mentions some statistical challenges in implementing the basic approach for a single indicator: 1) In spite of the structural similarity of all regions, differences in the population composition with respect to important risk factors are to be expected. This may lead to variation in crude prevalences at baseline. 2) Moreover, the populations may change over time, and they may change differently in different regions. 3) There may be global time trends or structural changes in the prevalence numbers. 4) There may be floor or ceiling effects. 5) To support final decisions, it might be desirable to reduce complex patterns in time trends across regions to a single number. This, however, may hide important data features influencing the final interpretation. 6) There is an interest in analysing an entire set of quality indicators. Consequently, we have to measure or phrase the intervention effect in a manner comparable across all indicators.

The purpose of this paper is to present the conceptual and statistical approach chosen to tackle these issues in the analysis of a single indicator, as well as the approach chosen to assess the overall evidence for an impact of the intervention. In addition, some further ideas and extensions are discussed.

## Methods

### Selection of control regions

The contract covers the Kinzigtal region, which is located in the Black Forest in Southwest Germany and home to about 70,000 inhabitants. This defines the intervention region.

The control regions were selected in a process described in detail in Additional file 1. As a first step, potential control regions were identified based on the following criteria, attempting to mimic basic features of the intervention region: geographically contiguous area; rural community or small to medium sized town (<50,000 inhabitants); river valley or active physicians’ network existing already in 2005. Regions with an integrated care contract lasting at least until 2015 were excluded. In a second step, the distribution of a series of structural indicators reflecting the social, economic and health services structures were compared among the 29 regions identified in the first step. The aim was to identify the regions most similar to the intervention region. In addition, the size requirement was tightened to include only regions with at least 35,000 inhabitants and to cover only towns with maximally 30,000 inhabitants. Immediate proximity to a hospital with maximum service level or to a major city, common border with Switzerland, and high internal heterogeneity were defined as further exclusion criteria. It was also taken into account that the overall number of insurees in the control regions and the intervention region should not exceed 500,000 in order to fulfill requirements on data protection. Finally, 13 control regions were identified that were regarded as showing a pattern of the structural indicators similar to the intervention region. Seven of the 13 control regions had an active physicians’ network.

We number the regions from 1 to *R*=13 for the control regions and use either *r*=0 or *KT* to designate the intervention region Kinzigtal (KT).

### Selection of indicators

The quality indicators to be analysed in this project were selected in a process described in [4]. In the end only indicators referring to a binary event in a specific population were selected. So in the classical terminology of a population-based quality indicator, it consists of a definition of a denominator, i.e. the size of a population of interest, and a numerator, i.e. the number of subjects in this population who experienced the event of interest. All selected indicators referred to populations defined on an annual basis, reflecting a common practice in defining indicators. Not all primarily selected indicators could be operationalized based on the available claims data, and some indicators could be operationalized in different manners [5]. Finally, 119 indicators were candidates for the statistical analyses. However, 13 indicators showed only very few events or non-events, such that the formal analysis described in this paper was not feasible. Among the 106 remaining indicators the prevalence ranged from 0.12% to 96.7% in the BW sample. As part of this selection process, the desired direction of a change in the indicator was also defined, i.e. whether an increase or a decrease is regarded as desirable. For five indicators, such a decision was not possible. Finally, it was decided whether it was reasonable to apply an indicator to subjects covered by a family doctor-centered healthcare contract or not.

### The data and the intervention(s)

The project makes use of the routine claims data of the AOK-BW. Of the 70,000 inhabitants of the Kinzigtal region, 33,000 are insured with the AOK-BW. Overall, the control regions cover about 452,000 insurees of the AOK-BW.

The shared savings contract and the regional integrated care model evaluated in the INTEGRAL project implied many different concrete interventions initiated by the management company. The interventions aimed at fostering patient self-management and shared decision-making (e.g. by supporting the use of individual treatment plans and goal-setting agreements) and at coordinating the care efforts across different sectors. A more detailed description can be found in [6]. It should be noted that many of these interventions aimed at an improvement at the system level and hence also patients not insured by the AOK-BW may have been affected. Effects in this patient population could of course not be evaluated in the INTEGRAL project.

### Time range

The time range included the years 2006 to 2015. It was originilly planned to include data from 2005, i.e. the year prior to the start of the intervention. However, it turned out that the data of 2005 were not of sufficient quality to be included in the analysis. The insufficient quality can be explained by the fact that the data warehouse of the AOK research institute (WIdO) was still in its start-up phase at that time.

### Data preprocessing

The indicators were operationalized based on the claims data of the AOK-BW. Details are described in [5]. Roughly speaking, for each calendar year from 2006 to 2015 we identified the population living in the intervention region or one of the control regions who were insurees of the AOK and satisfied the denominator definition of the indicator. Then for each member of this population it was determined whether the event of interest happened or not. More details are outlined in Additional file 2.

In addition, a 10% sample of the entire BW population of members of the AOK was drawn as described in Additional file 2. Excluding subjects living in the intervention region (but not those living in one of the control regions), an additional region – called the “BW region” in the sequel – was defined for each year. This was regarded as an alternative control region supplementing the control regions described above. However, when referring to “control regions” in the following, we do not include this additional region. When referring to region numbers, this region has the index *R*+1=14, but also the index “BW” is used. The 10% sample will be also used for the purpose of standardization.

For a single indicator considered in this project, the analysis population is defined as the union of all these populations, i.e. from the intervention region, the preselected control regions, and the BW region. Subjects could be followed over time, and hence the basic data can be described by random variables *Y*_*it*_, denoting the outcome of interest in subject *i* in year *t*. The outcome may not be defined for all years in the event a subject enters or leaves the denominator population. We implicitly assume in the following that for each year only the subjects included in the denominator population of the indicator are included in the analysis. In addition, the variable *R*_*it*_ denotes the region subject *i* was living in year *t*, which may change over time due to moving. The years are numbered from *t*=0 to *T*. The value of *T* was 9 for most indicators, but a few indicators could not be operationalized in the first years and covered a more narrow time range.

### Unit of prevalence trends

Independent of the type of event defining an indicator, we refer to the frequency of an event in the denominator population of a year as the (annual) prevalence. Time trends in prevalences, i.e. changes in prevalence over time, will build the main corner stone of the analytical approach. We decided to express trends as absolute differences over time, as stakeholders reading the analyses of single indicators can be expected to have some background knowledge – including an idea about the prevalence – such that they can interpret absolute differences. When later summarizing results across different indicators, we will change partially to an odds ratio scale to achieve a better comparability across indicators.

We decided to report trends in prevalence with respect to a five years interval to assist in the interpretation. Five years reflect a time period at which one typically expects to see an effect from a population-based intervention. We did not report annual changes, as such numbers tend to be small, and hence invite an overly pessimistic interpretation. Irrespective of this timescale, all trend estimates will be based on all years for which data was available. We further decided to report the five-year trends in percentage points instead of fractions between 0 and 1, again mainly to avoid over-pessemistic interpretations.

### Confounders

The comparison regions were selected with the aim of facilitating comparability with the intervention region. Nevertheless, we have to expect differences in the composition of patient populations with respect to variables such as age, gender, comorbidity status and socio-economic status (SES). These differences can also explain differences in the prevalence of indicators. Hence, it is a common approach in population epidemiology to adjust for such differences by appropriate standardization [7, 8].

This is typically done in order to allow a fair comparison across regions with respect to the prevalence. This is to some degree also relevant in our context, as comparability of prevalences across regions at baseline facilitates the interpretation of trends. However, our main interest lies with the time trends themselves. It is thus relevant for us to address confounding with respect to time trends. A typical example would be age demographics with differences in the growth of elderly populations across regions. This will increase the variation in trends for any indicator with the probability of an event increasing with age, e.g. the population prevalence in diabetes. This may even bias the estimation of intervention effects, if the intervention region has a specific speed.

A further crucial point may be that the intervention itself may change the distribution of covariates in a way, such that adjustment introduces a bias. For example, the intervention may reduce comorbidity, and then adjustment for comorbidity punishes the intervention region for this. Or the intervention may improve the documentation of the comorbidity level, and hence the population in the intervention region seems sicker on average than in reality, giving the intervention region an unwarranted advantage when adjusting. To avoid such problems, we will use the insuree’s comorbidity level at the time of entering the study population instead of using the actual value from each year.

The use of claims data restricts the possibility to define confounders. We make use of the following three potential confounders:

(i) age (in years)
(ii) gender
(iii) Charlson comorbidity index at study entry

In addition, we make use of the SES, operationalized by the German Index of Socioeconomic Deprivation (GISD, [9, 10]). While not available at the individual level, the SES was available at the regional level of municipalities associations and could be assigned by postal code. The intervention region and the 13 control regions covered 1687 different post codes overall, such that on average 4518 subjects shared a postal code. For the study population the GISD varied between 4.76 and 8.72, where higher values stand for a more pronounced deprivation and hence a lower SES.

### Family doctor-centred healthcare

AOK-insurees in the federal state of BW are offered participation in a specific family doctor-centered healthcare program. Insurees enrolling into this program choose a fixed general practitioner (GP) as their family doctor. Unfortunately, for some health services, this implies a lack of claims data in these insurees, as they are covered by a general fee for the GP. Consequently, if an insuree signs such a contract, certain events will not be visible in the claims data. If the event definition of an indicator could have been affected by this, the patients with this type of contract were removed from the analysis population. This may introduce a biased selection, as insurees signing such a contract may be more (or less) healthy than the general population. However, by adjusting for the above mentioned confounders, we can at least limit a corresponding selection bias.

### Relevance limits

The precision at which prevalence trends can be estimated varies highly from indicator to indicator due to differences in the size of the denominator population and the prevalence. Hence it would be highly misleading to regard statistical significance as the solely relevant criterion. The magnitude of the observed trend differences should be taken into account, too.

Originally, the idea was to ask the medical experts involved in the development of the indicators to also define limits for clinical relevance. This turned out to be not manageable due to the inherent arbitrariness and also the large number of indicators involving very different specialities. We hence decided to use a numerical criterion. For this we first considered the range of potential improvement, taking the prevalence *π* in the BW sample in the initial year as a starting point. For an indicator with a desired increase in prevalence, the potential improvement was then defined as the difference between 100% and the prevalence. For an indicator with a desired decrease in prevalence, it was defined as the difference between the prevalence and 0%. A consensus process within the research group resulted in setting the relevance limit as 10% of the potential improvement, as well as to require minimum 0.1 percentage point improvement. (Cf. [11] for a similar approach).

### The medical practice of a patient

For each patient in the analysis population of an indicator and for each calendar year, the health care provider who was likely responsible for the management of the patient was determined. This was assessed by an algorithm that, roughly speaking, identified the primary responsible provider (depending on the tracer diagnosis) based on the following criteria (in descending order): highest number of treatment quarters, contacts (days with services), and number of services billed. In case of ties, the provider with the first contact in the calendar year was chosen. If still a unique provider could not be identified, a random selection was made. In general, health care provider refers here not to a single individual, but to a practice, possibly constituted by several individuals. We refer to this as the practice of the patient. The precision of the assignment varies from indicator to indicator, as for some indicators it can be challenging to determine a responsible provider. This information will not be used in the main analytic approach, but only in sensitivity analyses and specific extensions.

### Main analytic approach

The analysis of a single indicator is based on the following five steps:

(i) estimating standardized annual region-specific prevalences *p*_*rt*_
(ii) estimating five-year time trends *θ*_*r*_ in each region
(iii) estimating the mean trend *μ*_*C*_ in the control regions and the standard deviation *σ*_*C*_ of the true trends across the control regions
(iv) computing key figures to assess the difference in trend in the intervention region relative to the control regions, in particular $\hat {\Delta }_{C} = \hat {\theta }_{KT} - \hat {\mu }_{C}$, i.e., the difference between the trend in the intervention region and the mean trend in the control regions, and a z-score *z* relating the observed difference $\hat {\Delta }_{C} $ to the standard deviation $\hat {\sigma }_{C}$ of the trends in the control regions
(v) verbal classification of the results as a strong positive (or negative) hint, a regular positive (or negative) hint, a weak positive (or negative) hint, or inconclusive while taking the relevance of the magnitude of the difference *Δ*_*C*_ into account

In principle, the first four steps could be replaced by fitting one complex model integrating all steps. We preferred a step-wise approach, as it allowed us to achieve more transparency about the process from the original data to the final results. To increase this transparency, the numerical results from each step are visualized in a user-friendly manner. This is exemplified in the single indicator “Treatment with acetylsalicylic acid (ASA)” in the sequel. This indicator aimed at the percentage of coronary heart disease patients who received a prescription for ASA within the last 12 months. Further examples will be presented later.

All statistical computations were performed with Stata 15.

### Estimating standardized prevalences

Estimates of the annual region-specific prevalences were based on a direct standardization to the 10%-sample of BW. This was achieved by 1) using the maximum likelihood principle to fit a logistic regression model to the data from all regions and time points describing the individual probability of an event as a function of region, calendar year and the four potential confounders, 2) applying the derived (year and region specific) prediction rule to all subjects in the BW sample, and 3) averaging over all the individual probabilities.

In the logistic regression model it is assumed that all confounders have the same effect in all regions and at all time points, but we allow a region and time specific intercept. Due to the large sample size we allow for gender specific effects of age, comorbidity and GISD and also potential non-linear effects of age and comorbidity.

The logistic regression model considered reads

$$\begin{array}{@{}rcl@{}} logit \ P_{\alpha,\beta} \! \! & \! \! \! \!& (Y_{it}=1 | X_{it}) = \alpha_{R_{it}t} \\ && + \ \beta_{gender} 1_{\{gender_{i}=1\}} + \beta_{gisd}^{gender_{i}} gisd_{it} \\ && + \ f^{age}_{\beta_{age}^{gender_{i}}}(age_{it}) + f^{comorb}_{\beta_{comorb}^{gender_{i}}}(comorb_{it}) \end{array} $$

with *α* and *β* denoting parameter vectors and *f* functions with self-explanatory indexing. The gender specific parametrization is dropped if there are <100 events (or non-events) in one gender. For the gender-specific age effects we chose $f^{age}_{\beta }(x)$ as restricted cubic splines (also called natural cubic splines) with *k* knots. *k* is chosen as the difference between the 99% and 1%-ile of the gender specific age distribution, divided by 10 and rounded up to the next integer while allowing a minimum value of 2 and a maximum value of 4. The spline knots are placed at the 10th, 50th and 90th percentile if *k*=3, and at the 5th, 35th, 65th and 95th percentile if *k*=4. In the case *k*=2 the function $f^{age}_{\beta }(x)$ is linear, so the knot placement does not matter. To increase the numerical stability, the splines are based on the mean centered version of the variable age. For the gender-specific effect of comorbidity we consider an alternative approach taking the typical skewed distribution of comorbidity indices into account. We use the function

$$f_{\beta}^{comorb}(x) = \alpha + \left(\sum\limits_{l=0}^{L-1} \beta_{l} 1_{\{x=l\}} \right) + \beta_{L}(x-L) 1_{\{x \geq L\}}. $$

Here *L* is chosen as the upper 90%-ile of the gender-specific distribution of the integer-valued variable *comorbidity*, rounding down but choosing at most the second largest observed value. This way it is ensured that the linear part is based on at least 10% of the available observations and that there are at least two different values among these observations. Furthermore, if only two different values are observed in the variable *comorbidity*, *L* is set to 0 and the model reduces to a simple linear model. The variable is dropped from the model if it is constant. Since there is already an explicit gender effect in the model, we set *α*=0 to ensure identifiability.

The standardized prevalences are obtained as $ \hat {p}_{rt} = $

$$\begin{array}{@{}rcl@{}} \frac{1}{|S^{BW}_{t}|} \sum\limits_{i \in S^{BW}_{t}} \Lambda (\! \! & \! \! \! \! &\hat \alpha_{rt} + \\ && \hat \beta_{gender} 1_{\{gender_{i}=1\}} + \hat \beta_{gisd}^{gender_{i}} gisd_{it} + \\ && f^{age}_{\hat \beta_{age}^{gender_{i}}}(age_{it}) + f^{comorb}_{\hat \beta_{comorb}^{gender_{i}}}(comorb_{it})), \end{array} $$

with $S_{t}^{BW}$ denoting the individuals in the BW sample at time *t* and

$$\Lambda(x)=logit^{-1}(x)=\frac{1}{1+e^{-x}}. $$

The standard error of each standardized prevalence is obtained by application of the delta rule. Standard errors for the parameter estimates of the logistic regression model are based on robust standard errors taking clustering within an individual over time into account. Due to the large sample size of the BW sample, the full application of the delta rule was not possible and a Monte Carlo approximation was used as outlined in the Appendix.

**Fig. 1:**
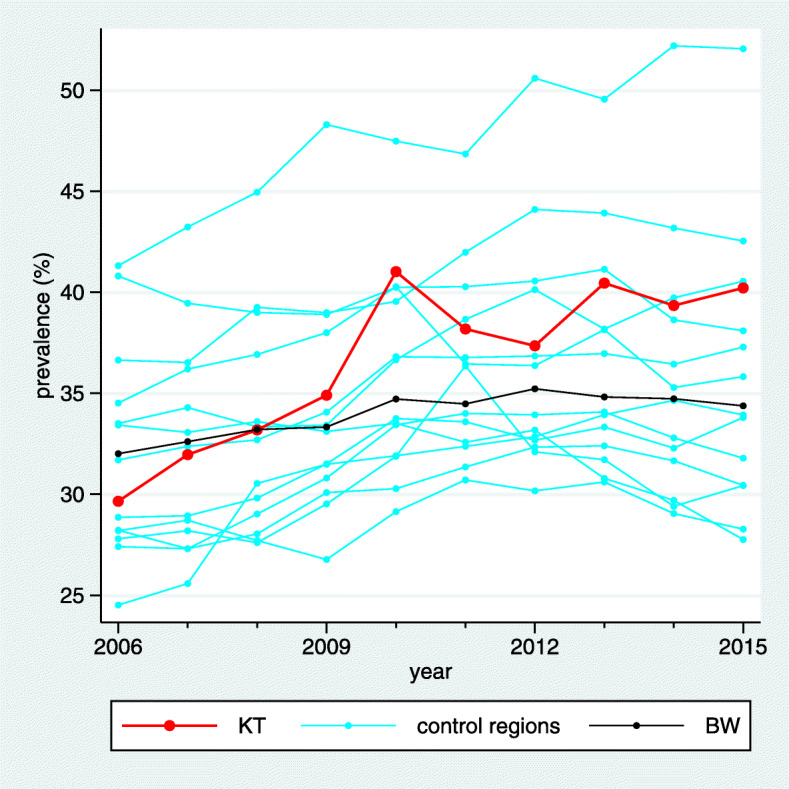
Visualization of the estimated standardized prevalences in a line plot for the indicator “Treatment with acetylsalicylic acid (ASA)”, for which high prevalences are desired. In the BW region we can observe a slight upwards trend and the trends vary substantially across the control regions. In the intervention region “Kinzigtal” (KT) the prevalence starts below the BW prevalence, but is distinctly above the BW prevalence at the end of the observation period

The estimated standardized prevalences are visualized by line plots (Fig. 1) distinguishing the intervention region, the control regions, and the BW region by different colors. No confidence intervals are shown in these plots to avoid an overcrowded visualization.

### Estimating time trends

If one could be sure that a clear linear trend were visible in the prevalences within each region, assessment of a trend would be rather straightforward. However, we have to anticipate that for some indicators there may be a non-linear overall trend, for example a flattening decrease/increase or a structural change due to administrative reasons. In such a situation, the trend of interest is the trend on top of the common pattern across all regions, i.e. how a region is developing compared to the overall trend. For example, when leaving aside the general trend, a prevalence trend of -0.01 over 5 years for a region should reflect that the prevalence decreased by one percentage point faster in five years as compared to the general trend. We refer to this definition of a trend as the “on-top-trend”, which is used independent of the observed pattern in the general trend. However, as on-top-trends do not reflect a general linear trend, they do not coincide with the trend visible in the inspection of the line plots mentioned above. To avoid this conflict, an estimate of the overall linear trend is added.

To obtain estimates for the on-top-trend $\tilde \theta _{r}$, we fit a meta-analytic model of the type

1 $$\begin{array}{@{}rcl@{}} \hat{p}_{rt} &=& \alpha_{t} + \beta_{r} + \tilde \theta_{r} t + \gamma_{rt} + \epsilon_{rt} \\ && \text{ with } \gamma_{rt} \sim N(0,\tau) \text{ and } \epsilon_{rt} \sim N(0,\hat \sigma_{rt})  \end{array} $$

with the side conditions $\sum _{t} \alpha _{t}=0$ and $\sum _{r} \tilde \theta _{r}=0$. Here *τ* denotes the unexplained variation across regions and time points (estimated in fitting this model), whereas $\hat \sigma _{rt}$ denotes the previously obtained estimate of the standard error of $\hat {p}_{rt}$ for *r*=0,...,*R*+1 and *t*=0,...,*T*, which is plugged in.

Estimates of the 5-year trends *θ*_*r*_ are then obtained by adding an estimate of the (linear) overall trend and multiplication with 5, i.e.

$$\hat{\theta}_{r} := 5 (\hat{\tilde{\theta}}_{r} + \hat{\theta}) $$

with $\hat {\theta }$ derived as the ordinary least squares (OLS) estimate from fitting the model

$$\hat \alpha_{t} = \mu + \theta t + \epsilon_{t}. $$

Note that we have for $\hat {\theta }$ the explicit representation

$$\hat{\theta} = \frac{\sum_{t=0}^{T} (t-\bar t) (\alpha_{t}-\bar \alpha)}{\sum_{t=0}^{T} (t-\bar t)^{2}} = \frac{\sum_{t=0}^{T} t\alpha_{t}}{\sum_{t=0}^{T} (t-\bar t)^{2}}. $$

**Fig. 2:**
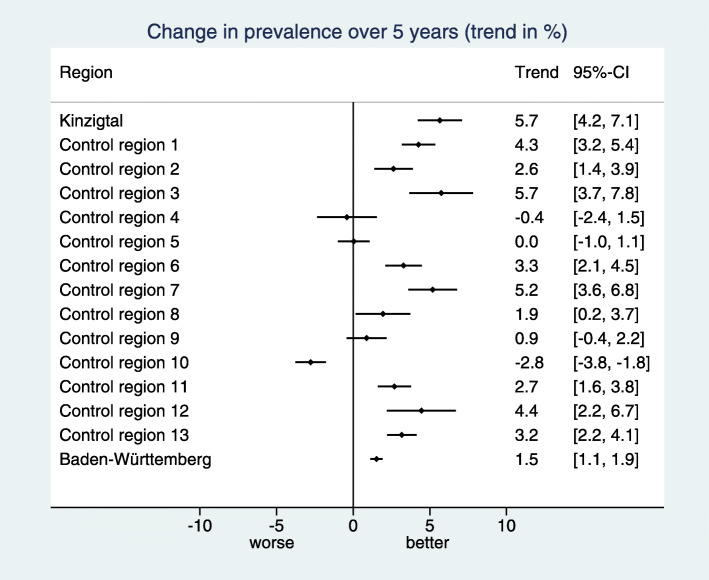
Visualization of the estimated trends per region in a forest plot for the indicator “Treatment with acetylsalicylic acid (ASA)". We can observe a positive trend for most control regions, i.e. also the control regions are able to move into the desired direction. However, the intervention region “Kinzigtal” shows together with one control region the most pronounced trend

Consequently, we have an explicit representation of $\hat {\theta }_{r}$ as a function of $(\hat {\tilde {\theta }}_{r})_{r=0,...,R+1}$ and $(\hat \alpha _{t})_{t=0,...,T}$, allowing us to compute easily its standard error based on the variance-covariance matrix of $(\hat {\tilde {\theta }}_{r})_{r=0,...,R+1}$ and $(\hat \alpha _{t})_{t=0,...,T}$. The results of this step – i.e. the region specific trend estimates with 95% confidence intervals – are visualized by a forest plot as shown in Fig. 2.

The choice of the structure of the model (1) is basically motivated by what can be achieved with standard software for meta regression – in particular the metareg command of Stata that we used. For this reason we could not incorporate the (estimable) correlations between the different $\hat {p}_{rt}$ (in particular within one region) and could not allow region specific variances for *γ*_*rt*_.

### Estimating the mean and standard deviation of the true trends across the control regions

Estimates for the mean *μ*_*C*_ and the standard deviation *σ*_*C*_ of the true trends are obtained by considering a meta-analytic model for the estimated trends, i.e.

$$\hat{\theta}_{r} = \gamma_{r} + \epsilon_{r} \text{ with }\gamma_{r} \sim N(\mu_{C},\sigma_{C}) \text{ and } \epsilon_{r} \sim N(0,\hat \sigma_{r}) $$

with $\hat \sigma _{r}$ denoting an estimate of the standard error of $\hat {\theta }_{r}$. We made use here of Stata’s metan command using the random option, implementing the method of [12].

### Computing key figures

The first key figure of interest is the difference between the trend in the intervention region and the mean trend in the control regions:

$$\Delta_{C} = \theta_{KT} - \mu_{C} $$

This informs us about the magnitude of the difference, and allows us in particular to judge the relevance. However, this does not address the question of which degree the difference can be regarded as exceptional or whether it is within the variation of trends to be expected when considering small local regions. To address this question, we relate the observed difference to the standard deviation of the trends in the control regions in the spirit of a z-score:

$$z = \frac{\theta_{KT} - \mu_{C}}{\sigma_{C}} = \frac{\Delta_{C}}{\sigma_{C}} $$

As a third supportive figure we consider the difference between the trend in the intervention region and the trend in the BW-region:

$$\Delta_{BW} = \theta_{KT} - \theta_{BW} $$

This is mainly included to allow a comparison with previous analyses based on a comparison with figures from BW [13]. The expectation is that the estimates of *Δ*_*C*_ and *Δ*_*BW*_ are similar. Discrepancies may remind us that the control regions differ substantially from the whole federal state of BW, which has to be taken into account when extrapolating effects of the intervention to the whole state.

For all three key figures we report estimates and confidence intervals. For the differences we also report *p*-values for a test of the null hypothesis of no difference. Confidence intervals for *Δ*_*C*_ and *Δ*_*BW*_ are computed as Wald-type intervals assuming independence between $\hat {\theta }_{KT}$ and $\hat {\mu }_{C}$ and between $\hat {\theta }_{KT}$ and $\hat {\theta }_{BW}$, respectively. Confidence intervals for *z* are based on Fieller’s method assuming independence between $\hat {\Delta }_{C}$ and $\hat {\sigma }_{C}$ [14].

Conceptually, the assumption of independence between the different $\hat {\theta }_{r}$ can be justified by the fact that they reflect mainly the data from the different regions, i.e. non-overlapping sources of information. However, as they are based on fitting one model, slight correlations cannot be excluded. Independence between $\hat {\Delta }_{C}$ and $\hat {\sigma }_{C}$ may be justified by the general result on independence between estimates of fixed effect parameter and random effect variances in mixed models.

**Fig. 3:**
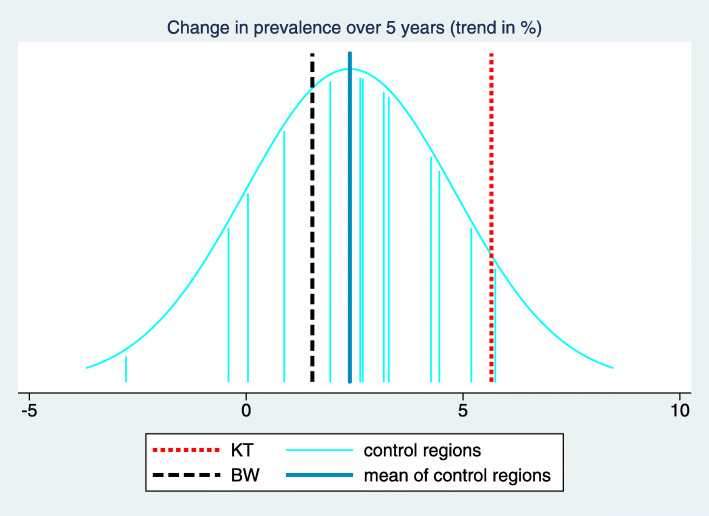
Visualization and tabulation of the key figures for the indicator “Treatment with acetylsalicylic acid (ASA)". We can observe that the trend estimate of the intervention region “Kinzigtal” is at the upper bound of the trend estimates of the control region. However, several other control regions have only a slightly less pronounced trend, and we obtain consequently a z-score of 1.35 only. The difference of the trend in the intervention region to the mean of the trends in the control regions is 3.27 with a confidence interval from 1.31 to 5.22. This is similar to the difference to the BW region, as the trend in the BW region is rather close to the mean of the trends in the control region

In order to facilitate the understanding of the background of these key figures, we suggest a two-dimensional visualization of all trend estimates and the estimated mean and standard deviation of the true trends in the control regions. This way the complete input used in computing the key figures becomes visible. In this visualization (Fig. 3) the x-axis refers to the trend and each trend estimate is reflected by a vertical line. Different colors are used for the control regions, the intervention region and the BW region, respectively. $\hat {\mu }_{C}$ and $\hat {\sigma }_{C}$ are transformed to a normal density superposed over the whole graph. $\hat {\Delta }_{C}$ and $\hat {\Delta }_{BW}$ are then visible as differences between lines of different colors, and $\hat z$ corresponds to the relative position of $\hat {\theta }_{KT}$ to the density function reflecting the variation of trends in the control regions.

### Verbal classification of the results

To come to a final conclusion about a potential specific role of the intervention region with regard to a single indicator, it is definitely not sufficient to look at the *p*-value to reject the null hypothesis *H*_0_:*Δ*_*C*_=0. This would ignore the relevance of the magnitude of the difference, and the relative position of the trend as expressed by the z-score. Since there is some freedom in how to summarize these different aspects in a final conclusion, we suggest a formal rule for verbalization of the results. This seems to be useful here, as we have to judge a large number of indicators, and a pre-specified formal rule helps to ensure a uniform, objective and transparent handling of all indicators. This can also serve as a basis for a formal assessment of the evidence about the role of the intervention across all indicators.

In the following, we denote with $\vec {\Delta }_{C}$ and $\vec {z}$ the directed version of the estimates $\hat {\Delta }_{C}$ and $\hat z$, i.e. in the case where a numerically negative trend implies an improvement in quality, we just change the sign of the estimates. With *b*_*l*_ we denote the lower bound of the 95% confidence interval for $\vec {\Delta }_{C}$ and with *b*_*u*_ the upper bound. *l*_*rel*_ denotes the relevance limit. In case of $\vec {\Delta }_{C}$ to be positive, we apply the verbal grading shown in Table 1. (The table is to be read sequentially: As soon as a condition is satisfied, the corresponding verbalization is applied.)

**Table 1:** The verbal grading used to classify hints

Verbalization	Trend	Z-score
strong positive hint	$ \vec {\Delta }_{C} > l_{rel} $ AND $b_{l}(\vec {\Delta }_{C}) > 0.5* l_{rel} $	$\vec {z} > 1.96 $ AND $ b_{l}(\vec {z})>1$
regular positive hint	$ \vec {\Delta }_{C} > l_{rel} $ AND $ b_{l}(\vec {\Delta }_{C}) > 0 $	$ \vec {z}>1.96$
weak positive hint	$ \vec {\Delta }_{C} > l_{rel} $	$ \vec {z}>1 $
inconclusive	otherwise	

Both the condition on the directed estimate as well as the condition on the directed z-score have to be fulfilled. The condition on the z-score, however, makes only sense if there is some evidence for a variation of the true trends across the control regions. Hence the condition is only applied if $\hat {\sigma }_{C}$ is above 0.001 and if the confidence interval for *z* is not degenerated (as it can happen when using the Fieller approach). In case $\vec {\Delta }_{C}$ is negative, we apply the corresponding scheme to define strong negative, regular negative, or weak negative hints or the choice of the verbalization “inconclusive”.

In the example considered in Figs. 1, 2 and 3 the desired direction is an increase in prevalence. The overall baseline prevalence in the BW sample is 32.0%, hence the relevance limit is 6.8%. The estimated trend difference $\hat {\Delta }_{C}$ between the intervention region and the control regions is 3.27%, so below the relevance limit. Consequently, the verbalization is “inconclusive”.

## Results

### Analysing an indicator with a global trend

**Fig. 4:**
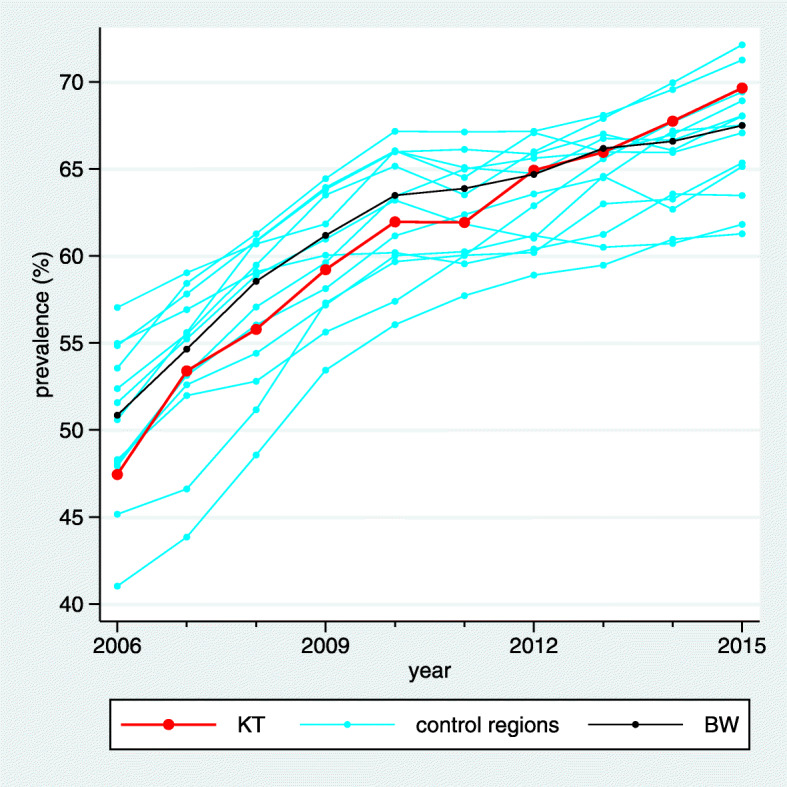
Visualization of the estimated standardized prevalences in a line plot for the indicator “Treatment with Statins I”

**Fig. 5:**
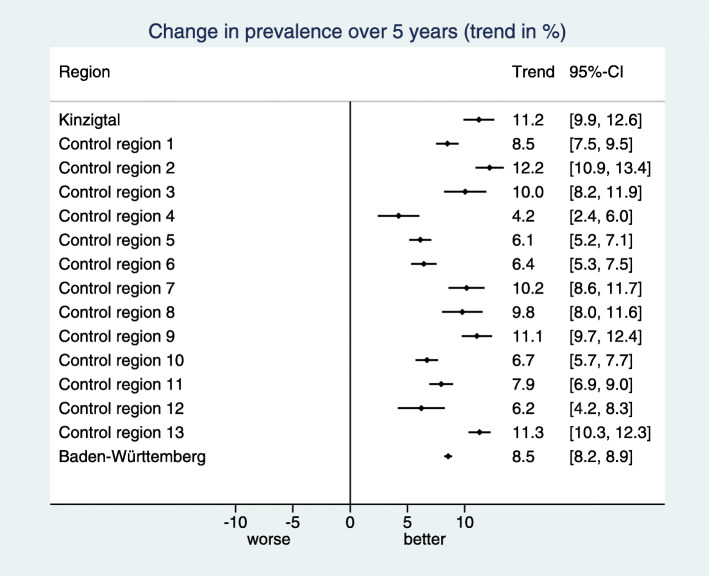
Visualization of the estimated trends per region in a forest plot for the indicator “Treatment with Statins I”

**Fig. 6:**
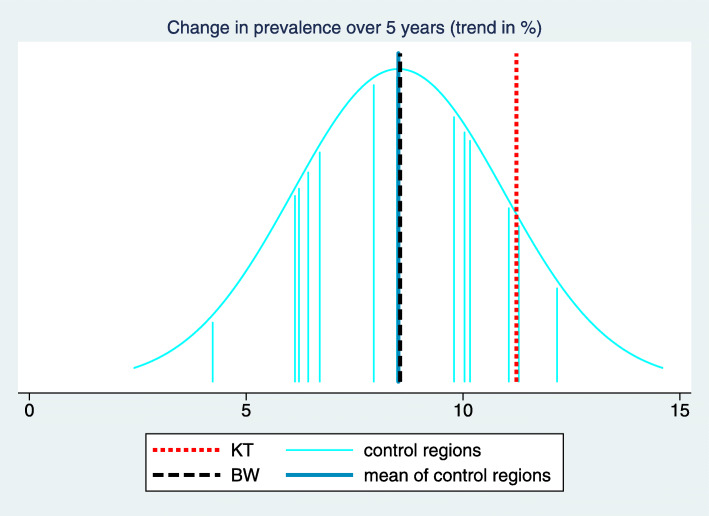
Visualization and tabulation of the key figures for the indicator “Treatment with Statins I”

It is well known that the use of statins in patients with coronary heart disease (CHD) has increased during the study period. This is also visible in Fig. 4 when considering the prevalence of the indicator “Treatment with Statins I” aiming at the prevalence of prescribing statins within the previous 12 months in CHD patients. In the intervention region the increase seems to be more pronounced than in the BW region, and in Fig. 5 we observe that it is also more pronounced than in many control regions. However, there are still several control regions with a similar (or even more pronounced) trend such that the z-score reaches only a value of 1.12 (Fig. 6). Moreover, the relevance limit is here 4.92%, and $\hat {\Delta }_{C}$ is below this value. Hence this result is verbalized as “inconclusive”.

### Analysing an indicator with a change point in the global trend

**Fig. 7:**
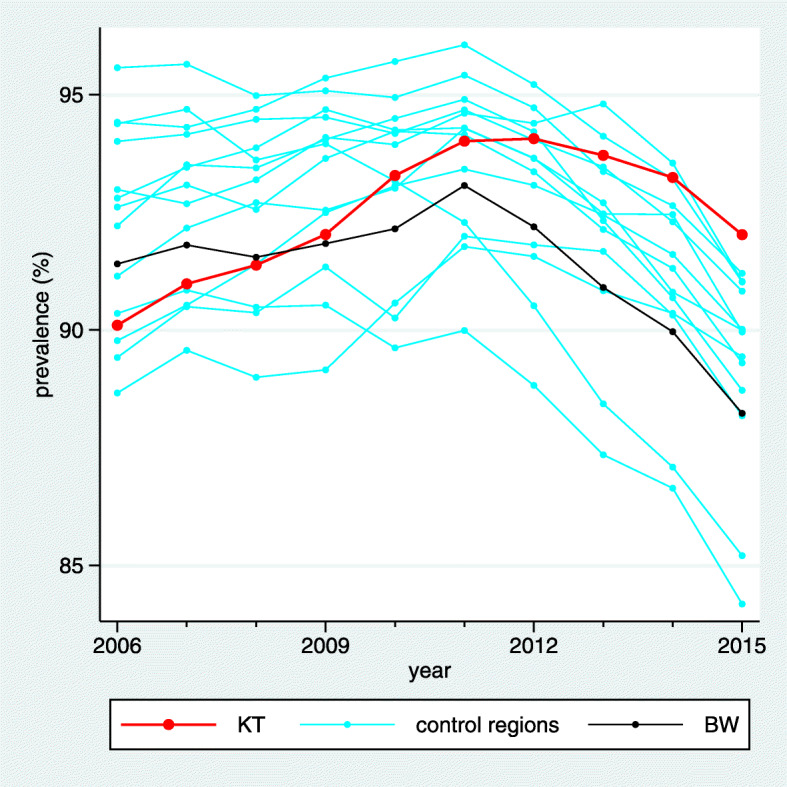
Visualization of the estimated standardized prevalences in a line plot for the indicator “Formulary concordant diabetes medication’

**Fig. 8:**
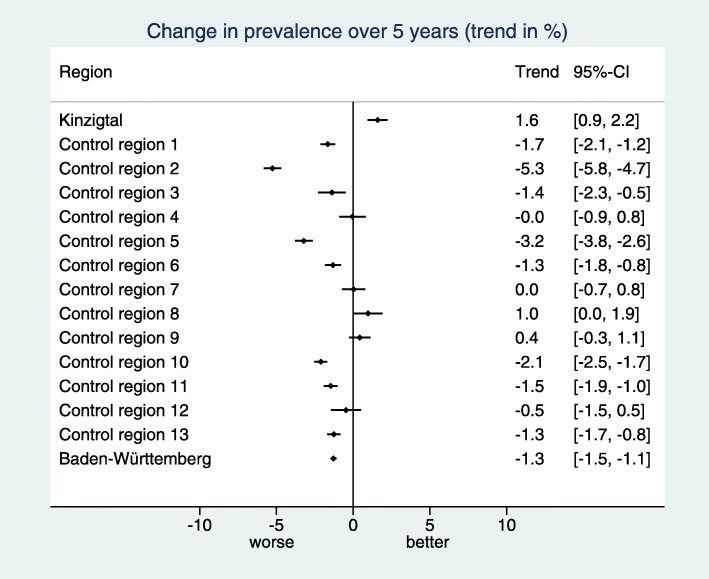
Visualization of the estimated trends per region in a forest plot for the indicator “Formulary concordant diabetes medication”

**Fig. 9:**
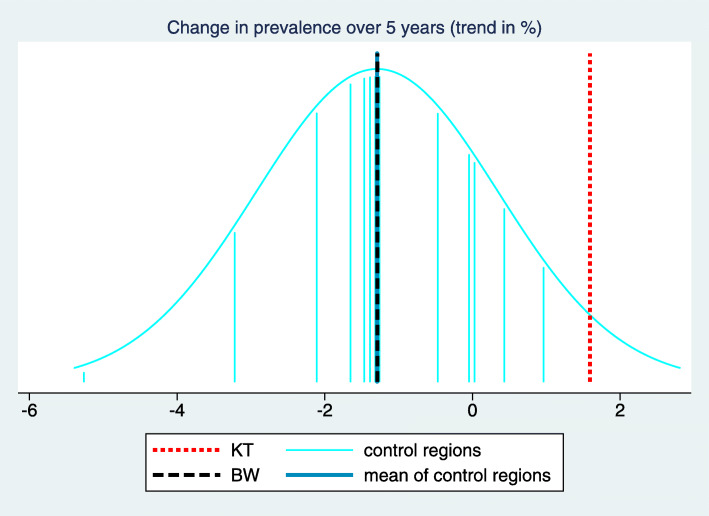
Visualization and tabulation of the key figures for the indicator “Formulary concordant diabetes medication”

During the study period, new drugs for the treatment of diabetes type II came into use. Hence an overall trend to more formulary concordant diabetes medication reversed around 2011 into a decreasing trend (Fig. 7). Also the intervention region followed this reversal, but in a less pronounced degree. Consequently, we observe in the intervention region a positive trend in contrast to most control regions (Fig. 8). Actually, the trend is higher than in all control regions (Fig. 9), resulting in a z-score of 1.76. Since the baseline prevalence of this indicator in the BW sample is already as high as 91.4%, the room for improvement is limited. The relevance limit is 0.86, and the lower bound 2.88 of the confidence interval for *Δ*_*C*_ is above this value. Consequently, the requirements for a weak positive hint are fulfilled. However, the z-score is not above 1.96, and hence the requirements for a regular positive hint are not fulfilled.

### Analysing an indicator with a structural change

**Fig. 10:**
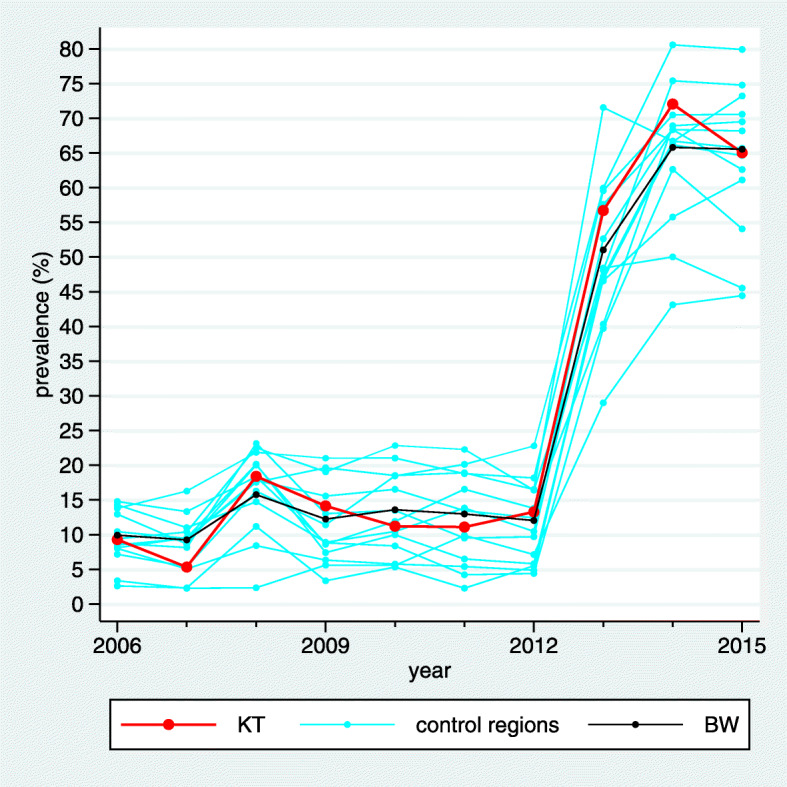
Visualization of the estimated standardized prevalences in a line plot for the indicator “GP-led geriatric assessment”

**Fig. 11:**
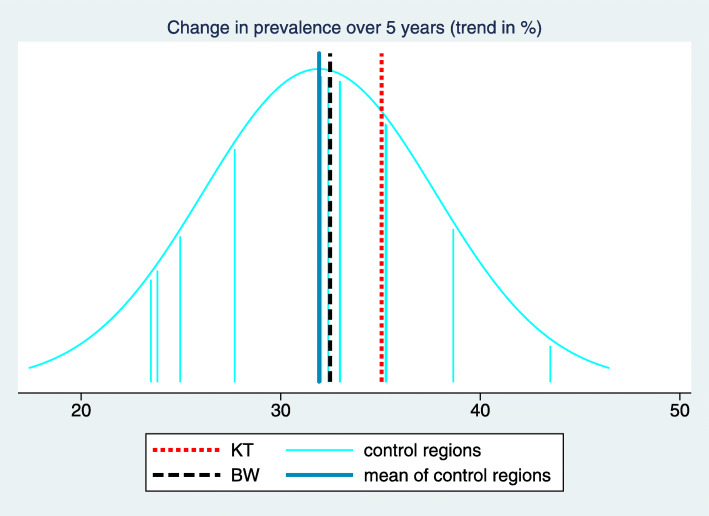
Visualization and tabulation of the key figures for the indicator “GP-led geriatric assessment”

Until October 2013, GP-led geriatric assessments required a corresponding additional certificate of the GP in order to be reimbursed. After this date, all GPs could expect reimbursement if the indication for such an assessment (defined by the presence of certain diagnoses) was given. This implied a substantial change in the corresponding indicator “GP-led geriatric assessment”, aiming at the prevalence of this assessment among all eligible patients. This is clearly visible in Fig. 10, and the intervention region follows this general trend as well as most control regions. Consequently, the trend in the intervention region is within the distribution of the trends of the control regions (Fig. 11), and the verbalization is just “inconclusive”.

### Assessing the overall evidence

Since the main analytic approach is applied to a large number of indicators, the question how to summarize the results in an adequate manner naturally arises. In particular, the question of the overall evidence for a specific role of the intervention region has to be addressed. In general we can try to summarize the evidence by computing a statistic for each indicator and to sum up these values. We consider here three approaches to compute such a summary statistic *S*:

(i) *S*_*hint*_ : Strong positive / negative hints are counted as +/- 5 points, regular hints as +/- 3 points, weak hints as +/- 1 points, inconclusive as 0 points, and the average is taken over all directed indicators.
(ii) *S*_*diff*_ : The directed estimates $\vec {\Delta }_{C}$ are transformed into log odds ratios comparing the two probabilities $\vec \pi + \vec {\Delta }_{C}$ and $\vec \pi $, (i.e. $ \log (\frac {\vec \pi + \vec {\Delta }_{C}}{1-(\vec \pi + \vec {\Delta }_{C}) }$ /$ \frac {\vec \pi }{1-\vec \pi }) $) and the average is taken over all directed indicators.
(iii) $S_{|\hat z|}$ : The average of $|\hat z|$ over all indicators.

The first two approaches aim to investigate whether the results in the intervention region (compared to the control regions) go on average either in the desired or in the opposite direction. The third approach looks only at whether anything special has happened in the intervention region, independent of whether it goes into the desired or the opposite direction. The approaches can be interpreted as a sequential procedure. The first approach takes the relevance of the differences into account, and requires a rather distinct effect for the single indicators. If this way some evidence for an overall positive or negative effect can be generated, the investigation can be stopped. However, if this way no effect is found, we may still obtain some evidence from the second approach, if there are many indicators with a (small) move into the desired direction – or if the opposite scenario holds. If this also fails, we can reach evidence in the third approach, if there are distinct trend changes (compared to the control regions) in many indicators, possibly in opposite directions. This could be interpreted in the way that the intervention had an overall impact, but sometimes in the desired and sometimes in the undesired direction.

In all three approaches it remains the question how to add inference statements to the computed statistics in a meaningful manner. Computing standard errors is not straightforward, as this has to take into account potential correlations across different indicators. Such correlations are not unlikely, as some indicators are conceptually closely related. Moreover, it is unclear which value the summary statistics should be compared to. For example, if we consider a null hypothesis of the type “There is no difference between the intervention region and the control regions for any indicator” (which can be operationalized in different manners), the expectation of *S* needs not to be zero. This follows from the fact that if the prevalence of an indicator is close to 0 or 1 (which has to be expected for a substantial number of indicators), it is not equally likely to obtain a positive or a negative hint of the same degree under such a null hypothesis. Sometimes, the requirements on relevance may even make it impossible to obtain a hint in one direction.

**Fig. 12:**
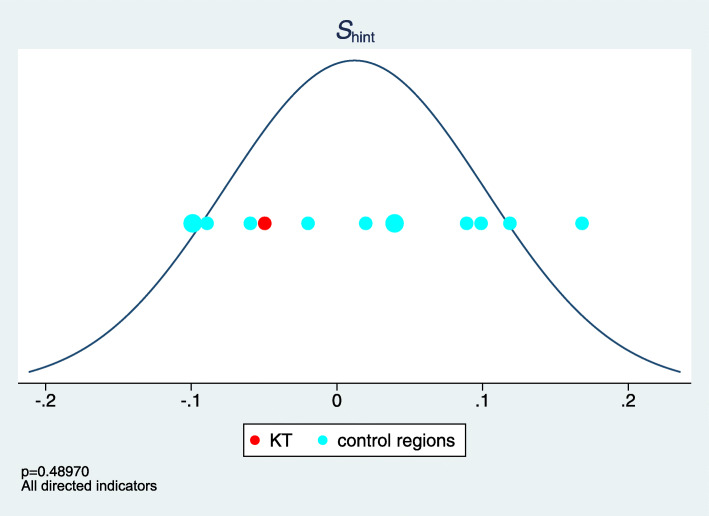
The values of the first summary measure for the intervention region and the 13 control regions and the approximating normal density curve

We hence suggest an alternative approach to obtain inferential statements. We can systematically exchange the role of the intervention region with any of the control regions and compute the value of *S* for any of these *R* scenarios. If we always observe a less extreme value in these alternative scenarios, then we have some evidence that the intervention region really plays a specific role. Formally, this can be regarded as a permutation test approach and a *p*-value can be computed by counting how often the originally observed value exceeds or falls below the values under the alternative scenarios. (In the third approach, only exceeding values are of interest.) However, based on *R*=13 control regions, this method leads to obtaining only a few different possible *p*-values. In particular, even if the intervention region is far away from all control regions, this does not imply a small *p*-value. We hence suggest to base the *p*-value on approximating the distribution of the 13 values under the alternative scenarios by a normal distribution. Subsequently all observed values are presented in a simple plot together with the approximating normal distribution, thus allowing the reader to judge the basis and adequateness of such a *p*-value. Figure 12 illustrates this approach using the first summary measure *S*_*hint*_ as an example.

This approach can also be used to analyze different subgroups of indicators, e.g. the program specific indicators. However, this rises additional multiplicity issues, which have to be addressed.

## Discussion

### Sensitivity and subgroup analyses

The overall analytical strategy of a single indicator is rather complex. Consequently, there may be concerns about specific assumptions or decisions made in the analysis process that may have an undesirable impact on the results. This motivates sensitivity analyses to investigate the stability of the results with respect to such assumptions and decisions. We consider in the sequel three different types of analyses that may be useful in this context.

### Technical sensitivity

This refers to assumptions and decisions made in the statistical modeling and computations. If such aspects are varied, we expect to see a very small impact on the results. Examples for technical sensitivity are

(i) Varying the size of the Monte Carlo samples described in the Appendix.
(ii) Varying the number of knots for the splines used to estimate the effect of age.
(iii) Consider practices instead of patients as clusters in computing robust standard errors.
(iv) Using the on-top-trends $\hat {\tilde {\theta }}_{r}$ as input in the meta analysis.
(v) Estimating *θ*_*r*_ by a meta regression within each region. These estimates are consistent for the same quantities, but less efficient, as variation due to an overall trend is not modeled. On the other hand, this approach allows a region specific error variance.

It would also be of interest to combine the different analytical steps described above into one model. In principle, this can be accomplished by incorporating the structure assumed for *p*_*rt*_ and for *θ*_*r*_, respectively, into the logistic model used to compute the standardized prevalences. In other words, $\alpha _{R_{it}t}$ is replaced with a random effects model for the trend. However, the parameters of such a model would refer to probabilities on the logit scale, and not to probabilities on a probability scale. Hence, they would be conceptually different. Alternatively, a generalized linear model with Bernoulli variance and identity link may be used. This may, however, be problematic with respect to modeling covariate effects correctly. In any case, it is not completely straightforward to combine the different steps in one model.

### Conceptual sensitivity

This refers to approaches that use the available data in a slightly different way. There is still the expectation to get similar results, which may be even more adequate. Examples for conceptual sensitivity analyses are:

(i) Restricting the analysis period to the first five years. In case the effect of the intervention was saturated after some time, this may result in more distinct effects. Otherwise, we would just loose efficiency due to a lower sample size.
(ii) Removing practices with prevalences already close to the desired prevalence over the whole period. Patients treated in these practices cannot improve any further, and this may mask intervention effects.
(iii) Using the intervention region instead of the BW sample for standardization. This may make the intervention effect more visible, if the intervention is best to a population close in composition to that of the intervention region.
(iv) Restricting the control regions to those with a regional physicians’ network. This comparison might be more fair, but less efficient.

### Subgroup analyses

Similar results in different subgroups of patients may underline that the intervention is really effective for all subjects. Differing results may inform us that the intervention is particularly helpful in certain subgroups. However, additional multiplicity issues may arise. Candidate variables for subgroup analyses are the confounders already available in all subjects, i.e. age, gender, comorbidity and SES.

### Identifying indicators with high likelihood for a specific role of the intervention region

When considering so many indicators, it is desirable to focus on the interpretation of those indicators with the strongest positive or strongest negative intervention effects, respectively. It is well known that just ordering the effects by size is a poor approach [15], as this invites over-interpretation of extreme results. In the last decades there has been substantial progress in developing methods to identify signals of interest in a more reliable manner based on the fact that a large number of indicators allows the estimation of the variation of the true effects. Consequently, the posterior probability to be above the clinical relevant threshold (cf. [16]) or characteristics of the posterior distribution of the rank can be considered [17] for each effect. However, the necessary computations are complicated by the fact that the effect estimates are not independent.

### Assessing potential acceleration and deceleration

It is one of the overall questions of the project, whether the effects of the interventions are long lasting. Some effects may vanish over time or they may become more distinct after some time. Investigating the stability of the trend may give an idea about this.

A simple approach is to consider within the intervention region estimates for the on-top-trend $\tilde {\theta }^{E}_{KT}$ in the first half of the time period, for the on-top-trend $\tilde {\theta }^{L}_{KT}$ in the second half of the time period, and for the difference $\Delta _{KT} = \tilde {\theta }^{L}_{KT} -\tilde {\theta }^{E}_{KT}$ as an expression of the acceleration/deceleration. Estimates for these quantities can be reported together with confidence intervals and *p*-values.

Estimates of these quantities can be easily obtained by replacing in model (1) the linear trend in the intervention region by a linear spline with a knot exactly at the middle of the observation period. This would again take into account that there might also be a change in the overall trend.

A verbal classification of the results is here more challenging, as there are a lot of possible patterns such as *vanishing, reversal, late start, acceleration* or *deceleration*. Furthermore, it is not straightforward to define rules to distinguish these patterns, in particular if relevance should also be taken into account.

### Assessing inter-practice variation

Improving quality indicators at the population level is the ultimate aim of any intervention of the type considered in this project. However, we can also investigate other aspects that may inform us how the intervention has (or has not) reached this aim. One of these aspects is the inter-practice variation. A high inter-practice variation is often regarded as an indicator of poor quality, as this may reflect a fundamental dissent among the health care providers about the optimal management of patients [18, 19]. Reducing inter-practice variation is hence also an aim of an integrated care model. However, such a reduction does not automatically imply an improvement in care; practices may also converge to a poor management strategy.

In any case it is rather simple to analyse trends in inter-practice variation with a similar analytic strategy. We just have to replace the estimates $\hat {p}_{rt}$ for the standardized prevalences in each region and year by estimates of the inter-practice variation. These can be easily obtained by fitting a logistic regression model with the practice as random intercept in each region and year with adjustment for confounders at the individual level. Thus, differences in the patient populations across practices are taken into account. Furthermore, by comparison with the control regions it can be taken into account that increasing or decreasing inter-practice variation in a region may also happen without an intervention.

Inter-practice variation can also occur with respect to the trends in the intervention region. Some practices may improve fast and some slow. This can be analysed with models including random intercepts and random slopes for each practice fitted to the data from the intervention region.

### Incorporating differences at baseline

It is natural that the prevalence of an indicator moves up and down within a region to some degree over time, either due to random variation or to temporal changes in the average patient management. Therefore, the intervention region may by chance have a relatively high or a rather low prevalence level at the baseline year *t*=0. This may lead to an increasing or decreasing trend over time, either due to regression to the mean or due to specific measures taken in the region to address the issue reflected by an indicator. In both cases a comparison with all control regions can become unfair, and it is desirable to compare the intervention region only to control regions with a similar level at baseline.

In the model (1) the parameter *β*_*r*_ reflects this baseline level for region *r*. So a naive implementation of this idea would be to restrict the comparison to control regions with an estimated baseline level $\hat \beta _{r}$ similar to $\hat \beta _{KT}$. This requires, however, defining some threshold distinguishing *similar* from *not similar*. This can be avoided by assuming a joint normal distribution for (*β*_*r*_,*θ*_*r*_)_*r*=0,…,*R*_ and to use this to predict *θ*_*r*_ based on *β*_*r*_. Then *μ*_*C*_ can be replaced by the predicted trend for a control region with a baseline value identical to that of the intervention region.

### Using automatically generated indicators

One limitation of the project is the restriction to a preselected albeit large set of quality indicators. These indicators may not cover all aspects of the health care system and hence there is a risk to overlook important trends. On the other hand, the claims data includes information on diagnoses, prescriptions, and medical procedures. They are based on well-established coding systems such as ICD, ATC, OPS, or EBM [20–23]. Hence we can also define indicators by defining events according to making a certain diagnosis, to prescribing a specific drug, or to receiving a certain medical procedure. Moreover, the hierarchical structure of the coding systems allows us to define meaningful groups of diagnoses, drugs or procedures, e.g. drugs with the same therapeutic intention. Hence thousands of events of interest can be defined for which time trends can indicate a change in patient management. However, the crucial point is to define the populations of interest. Just taking the whole population is possible and valid, but this may imply a lack of power in detecting trends. In principle, it is possible to also define risk populations in an automatic manner. For example, for a certain drug or drug group, we can identify all diagnoses that typically appear close to the prescriptions, and can define the population at risk as those suffering from such a diagnosis. Such an approach would be similar to existing data mining techniques in pharmaco-epidemiological data bases covering spontaneous reports of adverse events [24, 25].

### Code development

The analysis to be performed for a single indicator is rather complex. It involves the application of several statistical procedures in a sequential manner using the output of one method as input for the next method. The simultaneous application on 106 indicators (and the use of resampling procedures) makes it impossible to check the validity of each application by inspecting outputs and log files. Consequently, it was essential to develop code that was both robust and valid. The approach was hence split further into very small steps reflecting the application of single statistical procedures or certain data management actions. Each step was tested using input for which the desired output could be determined by independent means. A collection of such tests could be executed and compared to previous outputs in an automatic manner, allowing us to test the whole code after each development cycle.

The overall code could not be tested this way, as the desired output could not be determined by independent means. We hence generated data sets according to our overall model and tested the consistency of the resulting estimates by applying these to very large datasets. Furthermore, we explored the validity of the inference by checking the coverage of confidence intervals in simulation studies.

### Reporting

The application on 106 indicators (including descriptive tables to report basic properties of the data) and the systematic conduct of sensitivity analyses resulted in a huge amount of information. We hence generated a series of automated reports addressing different levels of interest. A first report (with nearly 10,000 pages) presented several items for each indicator. First, a summary of the results from the main analysis and all sensitivity analyses. Second, the complete results including some descriptive tables of the raw prevalences and the distribution of the covariates (stratified by region, year and both). Further reports focused on the acceleration or deceleration of trends, the analysis of the overall evidence, or provided an overview of all indicator specific results using tables and graphs presenting the numbers generated in a manner facilitating comparisons of interest. All these reports are available for the public (on request) at https://www.pmvforschungsgruppe.de/projekte/integral.html.

## Conclusions

The methodological approach developed in this project addresses general challenges in the evaluation of integrated care models such as accountable care organizations. This latter model for delivery and payment of health care has attracted much attention in the US [26] but also in Europe [27]. Recommendations for the evaluation of accountable care organizations were already developed some years ago [28, 29]. Since randomization is rarely feasible when introducing accountable care organizations, comparing patient groups exposed and unexposed to the new model in an observational pre-post design with adjustment for potential confounders can be regarded as providing the best available evidence [26]. This approach can be also called a difference-in-difference approach [30, 31]. The methodology developed for this project can also be seen as a difference-in-difference approach with adjustment for potential confounders. However, we consider the specific situation of evaluating one region-specific accountable care organization. This specific situation made it necessary to take into account that regional time trends can happen by chance. A comparison with several control regions lacking such an organization made is possible to tackle this challenge.

At first sight, the idea to compare prevalence time trends in an intervention region with corresponding trends in control regions sounds like a rather standard epidemiological analysis. However, implementation of this idea was rather complex, when taking into account the need for adjustment and the need to assess a specific role of the intervention region. We believe that the approach presented here can handle the major statistical challenges: population differences (also over time) are addressed by standardization; we offer transparency with respect to the derivation of the key figures; global time trends and structural changes do not invalidate the analyses; the regional variation in time trends is taken into account in the final judgement of the intervention effect. The latter three points are hopefully well illustrated by our examples. And it is worth mentioning that we could indeed observe a regional variation in time trends for the majority of indicators.

The simultaneous application on over one hundred indicators added further complexity, both computationally and conceptually. Overall, the project demanded substantial intellectual as well as organizational efforts to ensure adequateness, validity and transparency – as described in this paper – just to prepare the first main publication. Funders and scientists may tend to prioritize projects that can address the question of interest within a fully established methodological framework and an existing computational environment, such that results and publications can be generated with limited efforts. However, certain questions of high relevance such as the one addressed in this project require more efforts, and we should continue to plan and execute projects with an advanced methodological complexity.

## Appendix: Monte Carlo approximation of the standard errors of the standardized prevalences

To obtain the standard errors of $\hat p_{rt}$, we make use of the fact that we can express $\hat p_{rt}$ as

$$\hat p_{rt} = \frac{1}{|S^{BW}_{t}|} \sum\limits_{i \in S^{BW}_{t}} \Lambda \left(\sum\limits_{l} \hat \beta_{l} z_{il} \right), $$

ignoring in the notation that *β*_*l*_ may depend on *r* and *t* and *z*_*il*_ may depend on *t*. Consequently, we have

$$\text{Var} \left(\hat p_{rt} \right) = \frac{1}{|S^{BW}_{t}|^{2}} \left(\sum\limits_{i \in S^{BW}_{t}} v_{i} + \sum\limits_{i \neq i'} c_{ii'} \right), $$

with

$$\begin{array}{@{}rcl@{}} v_{i} &=& \text{Var} \Lambda \left(\sum\limits_{l} \hat \beta_{l} z_{il} \right) \approx l_{i}^{2} \cdot \tilde v_{i} \quad\text{ (Delta method:} \text{Var} f(X)\\ &\approx& \text{Var}(X) \cdot f'(X)^{2}) \\ l_{i} &= &\Lambda' \left(\sum\limits_{l} \hat \beta_{l} z_{il} \right) \quad \text{ with } \Lambda'(\cdot) = \Lambda(\cdot)[1- \Lambda(\cdot)])\\ \tilde v_{i} &= &\text{Var} \left(\sum\limits_{l} \hat \beta_{l} z_{il} \right) = \sum\limits_{l, l'} C_{ll'} z_{il} z_{il'} \end{array} $$

and

$$\begin{array}{@{}rcl@{}} c_{ii'} &= & \text{Cov} \left(\Lambda \left(\sum\limits_{l} \hat \beta_{l} z_{il} \right), \Lambda \left(\sum\limits_{l} \hat \beta_{l} z_{i'l} \right) \right)\\ &\approx& l_{i} l_{i'} \cdot \text{Cov} \left(\sum\limits_{l} \hat \beta_{l} z_{il}, \sum\limits_{l} \hat \beta_{l} z_{i'l} \right) \,\,\,\,\, \text{ (Delta method)}\\ &=& l_{i} l_{i'} \sum\limits_{l, l'} C_{l, l'} z_{il} z_{i'l'} \end{array} $$

where $\phantom {\dot {i}\!}C_{l, l'}$ denotes the covariance between $\hat \beta _{l}$ and $\hat \beta _{l'}$, and we can obtain an estimate for $\text {Var}(\hat p_{rt})$ by plugging in the estimated covariances.

The full enumeration of all pairs of subjects is, however, cumbersome, so we make use of a Monte-Carlo-approximation, based on expressing the variance of $\hat p_{rt}$ as

$$\begin{aligned} \frac{1}{|S^{BW}_{t}|} \left(\frac{1}{|S^{BW}_{t}|} \sum\limits_{i} v_{i} + \left(|S^{BW}_{t}| - 1 \right) \frac{1}{\left(|S^{BW}_{t}|-1 \right) |S^{BW}_{t}|} \sum\limits_{i, i'} c_{ii'} \right), \end{aligned} $$

allowing us to replace the two averages by averages based on subsamples. So we draw a subsample *S*_1_ of size *n*_1_ of $S_{t}^{BW}$ and approximate the first average by

$$ \frac{1}{|S^{BW}_{t}|} \sum\limits_{i} v_{i} \approx \frac{1}{n_{1}} \sum\limits_{i \in S_{1}} v_{i} $$

and a subsample *S*_2_ of size *n*_2_ of order pairs of $S_{t}^{BW}$, and approximate the second average by

$$ \frac{1}{\left(|S^{BW}_{t}|-1 \right) |S^{BW}_{t}|} \sum\limits_{i, i'} c_{ii'} \approx \frac{1}{n_{2}} \sum\limits_{(i_{1},i_{2}) \in S_{2}} c_{i_{1} i_{2}}. $$

We choose *n*_1_=min(*n*,10000) and *n*_2_=min([*n*/2]^−^,20000), with $n=|S^{BW}_{t}|$.

## Supplementary Information

**Additional file 1** The selection of structurally similar control regions for the intervention region in the INTEGRAL study. This pdf document includes additional information on the selection of the control regions.

**Additional file 2** Constructing the 10% sample Baden-Württemberg (BW) and defining study populations. This pdf document included additional information on the process of constructing the 10% sample and the definition of study populations.

## Data Availability

The raw data of the project is not available due to data protection reasons.

## Abbreviations

AOK: Allgemeine Ortskrankenkasse (health insurance fund)
ASA: acetylsalicylic acid
BW: Baden-Württemberg (federal state in Germany)
CHD: coronary heart disease
CI: confidence interval
DRKS: Deutsches Register Klinischer Studien (German Clinical Trials Register)
GISD: German Index of Socioeconomic Deprivation
GP: general practitioner
KT: Kinzigtal (region in BW)
OLS: ordinary least squares
SES: socio-economic status
WIdO: Wissenschaftliches Institut der AOK (AOK research institute).
