# Correction to: Assessing the effect of a regional integrated care model over ten years using quality indicators based on claims data – the basic statistical methodology of the INTEGRAL project

**DOI:** 10.1186/s12913-022-07881-y

**Published:** 2022-04-11

**Authors:** Dominikus Stelzer, Erika Graf, Ingrid Köster, Peter Ihle, Christian Günster, Patrik Dröge, Andreas Klöss, Claudia Mehl, Erik Farin-Glattacker, Max Geraedts, Ingrid Schubert, Achim Siegel, Werner Vach

**Affiliations:** 1 Institute of Medical Biometry and Statistics, Faculty of Medicine and Medical Center, University of Freiburg, Freiburg, Germany; 2 PMV research group at the Department of Child and Adolescent Psychiatry, Psychotherapy and Psychosomatics, University of Cologne, Köln, Germany; 3 Health Services and Quality Research, Research Institute of the Local Health Care Funds (WIdO), Berlin, Germany; 4 Institute for Health Services Research and Clinical Epidemiology, University of Marburg, Marburg, Germany; 5 Institute of Occupational and Social Medicine and Health Services Research, University Hospital Tübingen, Tübingen, Germany; 6 Basel Academy for Quality and Research in Medicine, Basel, Switzerland

**Correction to: BMC Health Serv Res 22, 247 (2022)**

**https://doi.org/10.1186/s12913-022-07573-7**

Following publication of the original article [1], it was found that information on shared authorship was accidentally missing. The correct information is as follows: Achim Siegel and Werner Vach share the last authorship.

The author group has been updated above and the original article [1] has been corrected.
