# Supplementary material for: Assessing the effect of a regional integrated care model over ten years using quality indicators based on claims data – the basic statistical methodology of the INTEGRAL project
Source: BMC Health Serv Res. 2022 Feb 24;22:247. doi: 10.1186/s12913-022-07573-7 (PMC8867633; doi:10.1186/s12913-022-07573-7)
Supplement: Supplementary file 1 — Additional file 1 The selection of structurally similar control regions for the intervention region in the INTEGRAL study. This pdf document includes additional information on the selection of the control regions. [file 12913_2022_7573_MOESM1_ESM.pdf]

Additional File 1

The selection of structurally similar control regions for the intervention region in the INTEGRAL study

Achim Siegel and the INTEGRAL study group

This is Supplementary Material to the manuscript "Assessing the effect of a regional integrated care model over ten years using quality indicators based on claims data – the basic statistical methodology of the INTEGRAL project" published in the European Journal of Health Economics. (First author: Dominikus Stelzer, Institute of Medical Biometry and Statistics, Faculty of Medicine and Medical Center, University of Freiburg, Stefan Meier Str. 26, D-79104 Freiburg, Germany, email: stelzer@imbi.uni-freiburg.de)

It is well known that the way a control group is selected is of crucial importance for the validity of a study. In a randomised controlled trial, the random allocation of subjects to either intervention group or control group(s) is intended to ensure that possible confounders – both known and unknown confounders – are equally distributed between intervention and control group(s). If for any reason no randomised allocation is possible, the control group(s) should nevertheless be as similar as possible to the intervention group in important aspects in order to minimize possible confounding. Without randomisation such a quasi-experimental parallelisation of different groups is only possible with respect to known or suspected confounders – unknown and unsuspected confounders can of course not be parallelised just because they are unknown.

In the case of the INTEGRAL study, no randomised study design was feasible as the intervention region had been determined long before the beginning of the INTEGRAL study. For this reason, the selection of the control group in the INTEGRAL study required a relatively high effort as the control group should nonetheless be similar to the intervention group in important aspects. According to the study funding application and study protocol, "approximately 10 structurally similar control regions" within Baden-Württemberg (the southwestern German federal state (Bundesland)) were to be selected for the given intervention region; about half of the control regions were to consist of regions in which a regional network of doctors operated in 2005, as it was the case in the Kinzigtal region in 2005. For reasons of data protection, the total number of insureds' data records in the various control regions should not exceed 500,000 per year.

The selection process proceeded in two phases or stages. These are described in greater detail below.

First stage of the selection process

In the first stage, the following inclusion criteria were defined.

- Each control region should preferably form a geographically contiguous area.
- Since the intervention region consisted only of rural communities and small towns (each with less than 20,000 inhabitants), each control region must consist only of the

following forms of settlement:

- rural communities,
- small towns[1] (less than 20,000 inhabitants as of 31.12.2015[2]), and
- small medium-sized towns (20,000 to less than 50,000 inhabitants, preferably less than 30,000 inhabitants as of 31.12.2015).

- Each control region must consist of administrative units (towns, individual municipalities and associations of municipalities), for which separate and publicly accessible basic statistical data were available.
- Each control region must either be characterized by a river valley or be a region where a regional network of doctors was active in 2005. (The intervention region was characterized by both features.)

An examination of the annual population figures between 2005 and 2015 showed that the population in almost all of Baden-Württemberg's municipalities, towns and cities reached its maximum during the last quarter of 2015. Therefore we used 31.12.2015 as the reference date for all population figures.

At the same time, the following exclusion criterion was defined:

- Regions in which a regional network of doctors had concluded an integrated health care contract with a health insurance fund that ran until at least 2015 must be excluded.

In the search for regional networks of doctors (which resembled the network that was active in 2005 in the intervention region), we were guided by the online list of the *Agentur deutscher Arztnetze e.V.* (https://www.arztnetze.info/). For all networks covering a relevant part of a possibly matching control region, basic data were researched, such as legal form, date of foundation, goals, regular activities, focus of activities, number of partners/members, and possible contracts with health insurance funds or with the Association of Statutory Health Insurance Physicians.

Based on the above-mentioned inclusion and exclusion criteria, a total of 29 potential control regions were identified; 10 of these regions had a regional network of doctors active in 2005, while the 19 remaining regions were characterized by a river valley. The population of the 29 regions ranged between 13,000 and 137,000. At the end of 2015, approximately 2 million inhabitants lived in the 29 regions. With an estimated rate of AOK-BW-insured persons of 35% to 40% (of the total number of inhabitants), this would have meant that up to 800,000 insureds' data sets would have been expected for the 29 regions. The above-mentioned mark of 500,000 would thus have been clearly exceeded. In addition, there was in some cases great heterogeneity between the 29 regions, e.g. in terms of urbanisation (types of settlement), income per capita, unemployment, educational level and structural

---

[1] We adopt here and in the following the definitions of the Federal Office for Building and Regional Planning. According to these, a small town [in German: Kleinstadt] has less than 20,000 inhabitants, a medium-sized town [Mittelstadt] has 20,000 to less than 100,000 inhabitants, and a major city [Großstadt] 100,000 or more inhabitants. A small medium-sized town [kleine Mittelstadt] has 20,000 to less than 50,000 inhabitants, a big medium-sized town [große Mittelstadt] 50,000 to less than 100,000 inhabitants. (https://www.bbsr.bund.de/BBSR/DE/Raumbeobachtung/Raumabgrenzungen/StadtGemeindetyp/StadtGemeindetyp_node.html; last access: 20.02.2018).

[2] All population figures were taken from publicly available data of the State Statistical Office Baden-Württemberg [Statistisches Landesamt Baden-Württemberg], https://www.statistik-bw.de/. The reference date was always 31.12.2015.

features of regional health care. For these reasons we continued the selection process.

Second stage of the selection process

In the second stage of the selection process we reduced this heterogeneity by sharpening the inclusion and exclusion criteria.

In the second stage the following inclusion criteria were applied.

- Each control region must form a geographically contiguous area.
- Each control region must consist only of the following forms of settlement:
  - rural communities,
  - small towns (with less than 20,000 inhabitants as of 31.12.2015),
  - very small medium-sized towns (20,000 to less than 30,000 inhabitants as of 31.12.2015) with a rather small-town appearance (which was assessed using aerial photographs taken from Google maps).
- Each control region must have at least 35,000 inhabitants as of 31.12.2015. (The intervention region had about 69,000 inhabitants at that time.)
- Each control region must consist of administrative units (towns, individual municipalities and associations of municipalities), for which separate and publicly accessible basic statistical data were available.
- Each control region must either be characterized by a river valley or be a region where a regional network of doctors was active in 2005.

In the second stage of the selection process regions with the following features had to be excluded:

- Regions in which a regional network of doctors had concluded an integrated health care contract with a health insurance fund that ran until at least 2015
- Regions in the immediate vicinity of a maximum care hospital (The intervention region is also not located in the immediate vicinity of a maximum care hospital.)
- Regions in the immediate vicinity of a major city with at least 100,000 inhabitants (The intervention region is also not located in the immediate vicinity of a major city.)
- Regions on the border with Switzerland (We feared that a border location with Switzerland – and thus a high proportion of cross-border commuters – would bring about a different composition of the AOK-BW insureds in those regions.)
- Regions with a considerable internal heterogeneity, e.g. with very different income tax revenues per capita in different parts of the region (The intervention region was fairly homogeneous as to various socio-economic and administrative aspects.)
- Regions that differed considerably from the intervention region in terms of nine indicators (see below for details of these indicators)

Important social, economic and health care structure characteristics of the 29 regions were considered in form of the above-mentioned nine indicators. These were collected for each individual administrative unit (town, individual municipality or association of municipalities) of a region so that also the internal homogeneity or heterogeneity of a region was revealed.

In the following the nine indicators are listed in detail:

- Unemployment in the years 2005, 2006 and 2007, averaged over all three years; data source[3]: INKAR (for reasons why we used data from the years 2005-07 see below)
- Income tax revenue per inhabitant in 2005, 2006 and 2007, averaged over all three years; data source: INKAR (for reasons why we used data from the years 2005-07 see below)
- Commuter balance 2014[4]; data source: INKAR
- Proportion of foreigners as of 31.12.2015; data source: State Statistical Office Baden-Württemberg
- Percentage of employees subject to social insurance contributions without vocational qualifications, as of 31.12.2015; data source: State Statistical Office Baden-Württemberg
- Percentage of employees subject to social security contributions with a university degree, as of 31.12.2015; data source: State Statistical Office Baden-Württemberg
- Average distance to the nearest hospital in minutes by car; data source: INKAR
- Number of inhabitants per general practitioner in private practice as of 31.12.2015; data source: Association of Statutory Health Insurance Physicians Baden-Württemberg
- Number of inhabitants per contract physician/psychotherapist in private practice as of 31.12.2015; data source: Association of Statutory Health Insurance Physicians Baden-Württemberg

As to the first two (of the nine) indicators, we focused on the years 2005-07, i.e. the beginning of the study period, for the following reason: (a) A short analysis showed that correlations between unemployment and life expectancy at birth (2014) in the different counties of Baden-Württemberg reached their maximum when unemployment data of 2005-07 were chosen – whereas correlations were lower when unemployment data from later years of the study period (i.e. 2008-15) were used. In other words, unemployment data taken from the beginning of the study period seemed to be the most relevant for population health (indicated by life expectancy 2014; 2014 was the most recent year for which life expectancy data were available at the time when we selected the control regions for the INTEGRAL study.) As to income tax revenue data, the reason why we focused on the years 2005-07 was similar. Correlations between income tax revenue per capita and life expectancy (2014) in the different counties and cities of Baden-Württemberg reached their maximum when income tax revenue data of 2005-07 were used. Correlations were lower when unemployment data were chosen from later periods.

Decisions about sufficient similarity between control regions and the intervention region and

[3] Three publicly available data sources were used to obtain the indicator values: 1) State Statistical Office Baden-Württemberg [Statistisches Landesamt Baden-Württemberg] (https://www.statistik-bw.de/), 2) INKAR database of the Federal Institute for Research on Building, Urban Affairs and Spatial Development [Bundesinstitut für Bau-, Stadt- und Raumforschung] (www.inkar.de), and 3) the 2016 Care and Quality Report of the Association of Statutory Health Insurance Physicians Baden-Württemberg [Kassenärztliche Vereinigung Baden-Württemberg] ("Die ambulante medizinische Versorgung 2016"; https://www.kvbawue.de/presse/publikationen/versorgungsbericht/).

[4] As to commuter balance and the remaining indicators in this list, the most recent data for the study period which were available in 2017 (i.e. at the beginning of the INTEGRAL study) were chosen. This meant we used commuter data as of 2014 and all other population data as of 31.12.2015.

about sufficient internal homogeneity of regions were based on visual inspection (by two researchers) of the distribution of the indicators. To illustrate a few 'exclusion cases' based on the above-mentioned indicators: we excluded e.g. the central part of the Rems Valley (located between Schwäbisch Gmünd and Waiblingen) because income tax revenue per inhabitant in 2005-07 was considerably higher in most parts of this region than in any part of the intervention region. We excluded e.g. the Bühlot-/Sandbachtal (located in the northern Black Forest around the towns Bühl and Bühlertal) because the proportion of employees with a university degree was, on the one hand, much higher than in the intervention region and, on the other hand, very unevenly distributed within the region itself (high internal heterogeneity). We excluded e.g. the Münster Valley (located in the southern Black Forest) because the numbers of inhabitants per general practitioner and per contract physician/psychotherapist in private practice were considerably lower than in the intervention region (which means that the average level of corresponding health services was expected to be much higher than in the intervention region). In addition, the region Münster Valley had to be excluded because of its small population (considerably below 35,000 inhabitants).

As a result, 13 control regions were finally selected; of these, 7 regions had a network of doctors in 2005. These 7 regions had a total of about 598,000 inhabitants at the end of 2015; the 6 regions without a doctors' network had a total of about 451,000 inhabitants. The population range of the 13 control regions was 36,000 – 131,000 inhabitants. The 13 control regions are listed below:

- Wiese Valley [Wiesental] (southern Black Forest)
- Elz Valley [Elztal] (central Black Forest)
- Rench Valley [Renchtal] (northern Black Forest)
- Acher Valley [Achertal] (northern Black Forest)
- Nagold Valley [Nagoldtal] (northern Black Forest)
- Tauber Valley [Taubertal] (northern Württemberg/northern Baden)
- Lower Jagst Valley [Unteres Jagsttal] (northern Württemberg)
- Lower Kocher Valley [Unteres Kochertal] (northern Württemberg)
- Bad Urach/Münsingen (County of Reutlingen, southeastern part)
- Donau Valley and Blau Valley [Donautal und Blautal] within the Alb-Donau County
- County of Sigmaringen
- Wangen/Leutkirch (County of Ravensburg, eastern part)
- Salem/Markdorf (Lake Constance County, central part)

Subsequently the appropriate postal codes were identified for all municipalities and towns in the intervention and control regions. In the statistical analyses and reports, individual control regions were pseudonymised.
