# Supplementary material for: Assessing the effect of a regional integrated care model over ten years using quality indicators based on claims data – the basic statistical methodology of the INTEGRAL project
Source: BMC Health Serv Res. 2022 Feb 24;22:247. doi: 10.1186/s12913-022-07573-7 (PMC8867633; doi:10.1186/s12913-022-07573-7)
Supplement: Supplementary file 2 — Additional file 2 Constructing the 10% sample Baden-Württemberg (BW) and defining study populations. This pdf document included additional information on the process of constructing the 10% sample and the definition of study populations. [file 12913_2022_7573_MOESM2_ESM.pdf]

Additional File 2

INTEGRAL – Constructing the 10% sample Baden-Württemberg (BW) and defining study populations

Ingrid Köster and the INTEGRAL study group

This is Supplementary Material to the manuscript "Assessing the effect of a regional integrated care model over ten years using quality indicators based on claims data – the basic statistical methodology of the INTEGRAL project" published in the European Journal of Health Economics. (First author: Dominikus Stelzer, Institute of Medical Biometry and Statistics, Faculty of Medicine and Medical Center, University of Freiburg, Stefan Meier Str. 26, D-79104 Freiburg, Germany, email: stelzer@imbi.uni-freiburg.de)

When drawing the sample and constructing the study populations, the following conditions and objectives had to be taken into account:

- For reasons of data protection, data for a maximum of n=500,000 additional AOK-BW insurees were to be transmitted to our study group, in addition to the complete data provided for AOK insurees of the Kinzigtal (KT) and control (CO) regions.
- Within each year, the selected insurees should be a random reference sample of the entire set of persons insured with AOK-BW.
- Over the years, individual insurees in the sample should be traceable to the same extent as AOK insurees in the KT and CO regions.

To approach this, the following steps were implemented at the AOK-BW:

- Among the set of all persons who were AOK-BW insurees at least once between 2005 and 2015, the number of those who did not live in any of the KT and CO regions was identified.
- As a result of the first step, it turned out that to make sure that an additional 500,000 AOK-BW insurees outside the KT and CO regions would be selected, a random sample of around 10% had to be drawn from the entire set of AOK-BW insurees.
- This selection was implemented as a proportionally stratified random sample, stratified for age and gender as of 31st December 2015, using the Oracle algorithm DMBS RANDOM.

Hence, an insuree whose data were provided to our study group should belong to the BW random sample or to the KT or a CO region, but could belong to the BW random sample as well as the KT or a CO region. This approach allowed us to determine study populations on a yearly basis in a comparable manner for each of the KT and CO regions, as well as for the intended 10% sample of AOK-BW insurees and for BW excluding KT. In particular, an insuree was included in a yearly study population if the person was

- insured with AOK-BW and living in the respective region for the entire calendar year *or*
- insured with AOK-BW and living in the respective region until death during the calendar year *or*
- insured with AOK-BW and living in the respective region from birth onwards during the calendar year.

Having drawn the original random sample from the set of all persons who were insured with AOK-BW at least once between 2005 and 2015, i.e. irrespective of the duration of insurance, allowed us to critically assess the selection effect of these criteria (data not shown). For the analysis of the single indicators, the study populations were then further restricted according to the denominator definition of the indicator.

Finally, standardised yearly indicator prevalences per region were produced based on the covariate distribution of the 10% BW sample, adjusting for four potential confounders as described in section 3.1 (Step 2). BW excluding KT, called “BW region” in the main paper, was used as a supplementary control region for comparison with KT as described in section 3.4.
